# Number of daily measurements needed to estimate habitual step count levels using wrist-worn trackers and smartphones in 212,048 adults

**DOI:** 10.1038/s41598-021-89141-3

**Published:** 2021-05-05

**Authors:** Jiali Yao, Chuen Seng Tan, Nicole Lim, Jeremy Tan, Cynthia Chen, Falk Müller-Riemenschneider

**Affiliations:** 1 Saw Swee Hock School of Public Health, National University of Singapore, Tahir Foundation Building (Block MD1), 12 Science Drive 2, #09-01v, Singapore, 117549 Singapore; 2 Policy, Research and Surveillance Division, Health Promotion Board, Singapore, Singapore; 3 Berlin Institute of Health, Charite University Medical Centre, Berlin, Germany

**Keywords:** Risk factors, Public health, Preventive medicine

## Abstract

Daily step count is a readily accessible physical activity measure inversely related to many important health outcomes. However, its day-to-day variability is not clear, especially when measured by recent mobile devices. This study investigates number of measurement days required to reliably estimate the weekly and monthly levels of daily step count in adults using wrist-worn fitness trackers and smartphones. Data were from a 5-month physical activity program in Singapore. The 5-month period was divided into 22 weekly and 5 monthly time windows. For each time window, we leveraged data sampling procedures and estimated the minimum number of measurement days needed to achieve reliable mean daily step count with intraclass correlation coefficients (ICC) above 80%. The ICCs were derived using linear mixed effect models. We examined both simple random and random consecutive measurement days and conducted subgroup analysis by participant characteristics and tracking devices. Analysis of weekly and monthly step count included 212,048 and 112,865 adults, respectively. Fewer simple random measurement days are needed than random consecutive days for weekly time windows (mean 2.5, SD 0.5 vs mean 2.7, SD 0.5; p-value = 0.025). Similarly, monthly time windows require fewer measurements of simple random days than random consecutive days (mean 3.4, SD 0.5 vs mean 4.4, SD 0.5; p-value = 0.025). Younger participants and those tracking steps via smartphones consistently required more days. Being obese was associated with more measurement days for weekly time windows. In sum, to obtain reliable daily step count level, we recommend at least 3 measurement days for weekly and 5 days for monthly time window in adults. Fewer days could be considered for adults age 60+ years, while more days are required when tracking daily step via smartphones.

## Introduction

Physical activity is a multi-dimensional behaviour key to health^1,2^. Its measurement is fundamental to its surveillance, promotion, and investigation of its relation to health^3^. Adequate physical activity measurements safeguard the strength of such practice and research. Over the past decades, device-based objective measurement approaches have been increasingly adopted aiming to reduce the errors and biases commonly found in self-reported physical activity, although many challenges persist^4,5^. One on-going debate centres around the number of measurement days needed to reliably estimate habitual physical activity^3–5^.

Daily step count is a common physical activity metric focusing on stepping behaviours. It presents an inverse dose–response relationship with important health outcomes according to the systematic review from the 2018 US Physical Activity Guideline Advisory Committee^6^. Rapid technological developments catalyse the widespread adoption of mobile and wearable devices able to track step counts. As a result, daily step count has become a readily accessible and feasible physical activity measure even in large-scale real-world settings^6,7^. However, daily step count exhibits substantial inter- and intra-individual variability^1,3^. To account for the day-to-day behavioural variability, researchers and practitioners usually take multiple days of measurement to capture an individual’s habitual step count level. Several studies, mostly conducted over a short duration, have investigated the number of days needed to measure habitual daily step count in adults reliably. A systematic review of reviews in 2018 found six such studies for different timespans. This systematic review concluded that a minimum of 2–4 days of measurement was required in older adults and 2–5 days in adults^8^. These findings differ from those of three other investigations found in the literature^9–11^ Due to the limited and heterogenous evidence available, it remains unclear how many measurement days are enough. In addition, these studies used either traditional Yamax pedometers or ActiGraph accelerometers worn on the hip^9–17^. It is unknown whether previous results can be generalised to step count measurement using the newly developed and widely adopted mobile tracking devices such as wrist-worn fitness trackers and smartphones. Whether participant characteristics influence the number of days required is another question that remains poorly understood.

Using daily step count collected objectively in a 5-month large-scale physical activity program, this study aims to examine the number of measurement days needed to reliably estimate the weekly and monthly levels of daily step count in adults. This study also investigates whether participant characteristics and device types affect the number of measurement days required.

## Methods

### Study design and participants

National Steps Challenge Season Three was a population-wide physical activity promotion program opened to the entire Singapore population aged 17+ years. The program was designed and implemented by the Health Promotion Board under the Ministry of Health, Singapore. Details of the program have been described elsewhere^18^. In total, 696,907 participants in Singapore registered for the program. Over 35 million participant-days of step count were collected objectively via wrist-worn fitness trackers or smartphone during the intervention period between 2017-10-28 and 2018-03-31^19^.

The current study includes a subset of the program participants who meet the following criteria: signing up for the program before 2017-10-28, providing valid demographic and anthropometric information, using only one tracking device model throughout the main intervention period, and recording step count for at least one full weekly study time window during the intervention period.

### Data sources

Demographic and anthropometric information was self-reported via the program App, including birthday, gender, weight, height, and the government-issued identification number, which can be used to differentiate Singaporean and foreigners. We considered this information valid if the participant provided identifiable nationality, gender, age above 17 years, weight between 30 and 300 kg, and height between 101 and 220 cm. Participants were categorised into four age groups and four weight status groups according to the Asian cut-offs of body mass index^20^. We extracted demographic statistics of the entire Singapore population aged 17+ years in 2018 from the United Nations website and Department of Statistics Singapore, as well as population statistics of body mass index from Singapore National Health Survey 2010^21–23^.

Daily step count was measured via participants’ preferred tracking devices and transferred to the program database via the program App. Step tracking devices in this study included four types of wrist-worn trackers offered free-of-charge by Health Promotion Board Singapore (HPB-Model 1-4), smartphones with built-in accelerometers (iPhone and Samsung phone), and self-purchased wrist-worn wearables (Fitbit and Actxa trackers). Actxa trackers are similar to those offered by Health Promotion Board Singapore, but they are commercially available at lower costs than Fitbit trackers^24^. While model details were recorded for devices from Health Promotion Board Singapore, this detail was not available for the remaining tracking devices. We identified Fitbit and Actxa devices by the brand names. Step count sourced from Apple HealthKit and Samsung Health App were categorised as iPhone and Samsung phone, respectively. According to unpublished data from 7356 individuals in Singapore Population Health Studies, collected between 2016 and 2018, only about 1% of the 1570 Apple HealthKit users tracked step count via Apple Watch^25^. Data of daily step count above zero step and collected during the main program intervention period were extracted for the main analysis.

### General approach

The program intervention period consists of either 22 weekly time windows (counting from the start date of the program, i.e., 2017-10-28) and or 5 monthly time windows (counting from 2017-11-01), a tally of 27 weekly or monthly study time windows. For each of the 27 time windows, we separately estimated the minimum number of measurement days required to obtain reliable mean daily step count level during the time window. The estimation was based on the outputs from a set of random data sampling procedures. Since not all participants recorded daily step count every day of the program intervention period, the analysis for each time window included participants who recorded daily step count on all the days of the week or month. We investigated the measurement days under two scenarios: with and without restricting the days to be consecutive.

### Sampling procedures for a study time window

**Figure 1:**
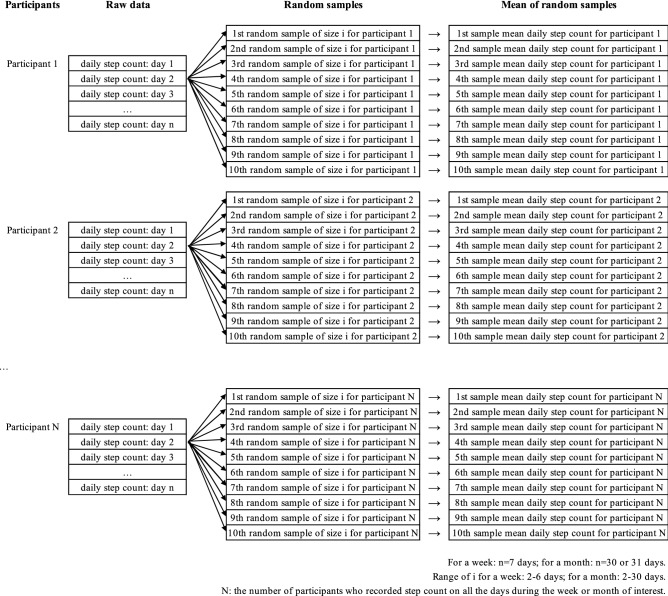
Data sampling procedures for a study time window and sample size of *i* measurement days.

For each time window, we drew random samples of different number of days from each participant’s complete daily step count data. The sample size, number of measurement days, ranges from 2 to 6 days for a weekly time window and 2–30 days for a monthly time window. Specifically, 2–29 days for the monthly window of November 2017; 2–30 days for the monthly windows of December 2017, January 2018, and March 2018; and 2–27 days for the monthly window of February 2018. The sample size was at least 1 day smaller than the size of the study time window, because drawing a sample with the size of the study time window is equivalent to directly using the complete data for the time window in this study. For each sample size, 10 samples were drawn for each participant, resulting in 10 sample mean daily step count for each participant for subsequent statistical analysis. Our preliminary analysis showed that 10 samples were sufficient to obtain stable estimates, and drawing extra samples did not improve the estimation. Figure 1 illustrates the procedure for one sample size *i* on one study time window. Sampling without replacement was used. Samples of random consecutive days and simple random days and represent the scenarios with and without restricting the continuity of the measurement days, respectively. While samples of simple random days were sampled directly, we obtained samples of random consecutive days by first randomly sampling the day one, then taking the following days in chronological order until the target sample size was reached. When there were no enough days of the time window left, the participant’s data were recycled. For instance, we treated a participant’s first day of a week as the “eighth” day of the week.

For the main analysis, samples were drawn independently for each participant. In other words, different calendar days of a week or month can be sampled for different participants.

### Outcomes

The primary outcomes were the minimum number of measurement days required, in a week and a month, such that the mean daily step count reaches a one-way random-effects intraclass correlation coefficients (ICC) of 80% or above, a commonly used cut-off for acceptable reliability^1,26^. The ICC represents the proportion of the variation in daily step count explained by the variation between participants. We leveraged data sampling and applied single score ICC with each sample mean daily step count as a score, instead of average score ICC with individual daily step count as a score. This is to avoid violating the statistical assumption that requires independent and identically distributed scores, an issue rarely considered in such studies before^1^.

Since ICC is a relative measure affected by inter-participant variability, we included the mean absolute percentage errors (MAPE) as secondary outcomes. The MAPE was to assess the intra-participant difference in the mean daily step count when the minimum measurement number of days were used compared to when the complete data including all the days of a time window were used. MAPE of a sample of *i* measurement days during a time window follows the formula:

$$ MAPE = \frac{{\left| {mean \,daily \,step \,count \,from\, i \,days^{\prime}\,data - mean \,daily\, step \,count \,from \,complete \,data } \right| }}{mean \,daily \,step \,count \,from \,complete \,data} \times 100\% . $$

### Statistical analysis

We summarised the participant characteristics by their step recording timeframe: whether they recorded for at least one complete week or one compete month. Where possible, we compared the characteristics of participants in this study with that of the Singapore population aged 17+ years using Chi-Squared tests.

We derived ICC using linear mixed effect models with participant-specific random intercepts. The dependent variable was the sample mean daily step count, ten sample mean daily step count values from each participant in each model. The models used independent within-group correlation matrix. For each study time window and scenario of measurement days, we conducted a series of models, resulting in a series of ICCs: one ICC value corresponding to one sample size (range of 2–6 days for a week and 2–30 days for a month). The smallest sample size with ICC over 80% was extracted, representing the minimum number of days needed to obtain mean daily step count reliably for the time window. Mean of MAPE in daily step count was computed using the corresponding minimum number of days. Subsequently, we summarised the minimum number of days and MAPE over the 22 weeks and 5 months. We also compared the difference in the minimum number of days required between using simple random days and using random consecutive days via Friedman's tests: one for the weekly estimate and one for the monthly estimate. The dependent variables was the minimum number of days required, the group variable was the sampling approach (simple random days vs. random consecutive days), and the block variable was the time window.

We conducted subgroup analysis by stratifying the above analysis by participant characteristics. Friedman's tests were used to compare the minimum number of days required (the dependent variable) between participant characteristic groups (the group variable), with the time windows as the block variable.

We performed several sensitivity analyses. First, we estimated the minimum number of days required for the settings when measurement days (in calendar day) are the same in all participants. This was done by first drawing ten sets of *i* random days then applying the ten sets of days to all participants each time. This is different from the main analysis where random days for different participants were drawn independently. Secondly, we conducted the analysis by only including the participants who recorded daily step count for at least a complete month and who did so for all 155 days of the program intervention period. As some participants recorded daily step count before the intervention, we repeated the main analysis using data collected prior to the intervention period.

All the analyses were conducted in R (version 3.6.1). R package ‘lme4’ (version 3.1-141) was used for linear mixed effect models, and R package ‘performance’ (version 1.1-24) for extracting ICC from the respective models.

### Ethics approval

Ethical approval for this study was obtained from the Institutional Review Board of the National University of Singapore.

### Declaration

All methods were carried out in accordance with relevant guidelines and regulations. Informed consent was obtained from all participants or, if participants are under 18, from a parent and/or legal guardian.

## Results

This study included 212,048 participants who recorded daily step count every day for at least 1 week (Table 1). Among them, 112,865 (53.2%) did so for at least one full month. On average, 95,673 (SD 8836) participants per weekly time window and 56,735 (SD 5878) participants per monthly time window provided complete daily step count data for analysis. Compared with the entire Singapore population, participants who recorded step count for at least one complete week consisted of more Singaporeans (65.5% vs. 59.1%), females (58.8% vs. 47.5%), and those with age 17–39 years (46.6% vs. 39.4%) and BMI 18.5 to < 23 kg/m^2^ (43.0% vs. 38.3%). The majority of participants recorded step count using smartphones (44.4%) and wrist-worn trackers offered free-of-charge by Health Promotion Board Singapore (43.2%).

**Table 1:** Characteristics of participants (N = 212,048).

Characteristics	Step count recording timeframe		Singapore population aged 17+ years, %
	At least one complete week	At least one complete month	
Total	212,048 (100.0)	112,865 (53.2)	
**Nationality**			
Singaporean	138,861 (65.5)	76,606 (67.9)	59.1
Foreigner	73,187 (34.5)	36,259 (32.1)	40.9
**Gender**			
Female	124,744 (58.8)	66,979 (59.3)	47.5
Male	87,304 (41.2)	45,886 (40.7)	52.5
**Age (years)**			
17–39	98,815 (46.6)	44,219 (39.2)	39.4
40–59	86,544 (40.8)	51,000 (45.2)	38.7
60–79	25,421 (12.0)	16,806 (14.9)	19.4
80+	1268 (0.6)	840 (0.7)	2.5
**Body mass index (kg/m**^**2**^)			
< 18.5	13,422 (6.3)	6989 (6.2)	6.4
18.5 to < 23	91,193 (43.0)	49,661 (44.0)	38.3
23 to < 27.5	74,481 (35.1)	40,174 (35.6)	32.3
≥ 27.5	32,952 (15.5)	16,041 (14.2)	23.0
**Participation of previous program season**			
No	116,470 (54.9)	56,200 (49.8)	
Yes	95,578 (45.1)	56,665 (50.2)	
**Step tracking device**			
HPB tracker 1	64,554 (30.4)	33,711 (29.9)	
HPB tracker 2	13,359 (6.3)	7924 (7.0)	
HPB tracker 3	4928 (2.3)	4015 (3.6)	
HPB tracker 4	8616 (4.1)	5179 (4.6)	
iPhone	49,298 (23.2)	23,250 (20.6)	
Samsung phone	44,902 (21.2)	24,437 (21.7)	
Fitbit	21,849 (10.3)	11,825 (10.5)	
Actxa	4542 (2.1)	2524 (2.2)	

Data are in N (%). All p-value < 0.001 by the Chi-Squared tests comparing the participant characteristics with the entire Singapore population aged 17+ years.

*HPB* Health Promotion Board.

**Figure 2:**
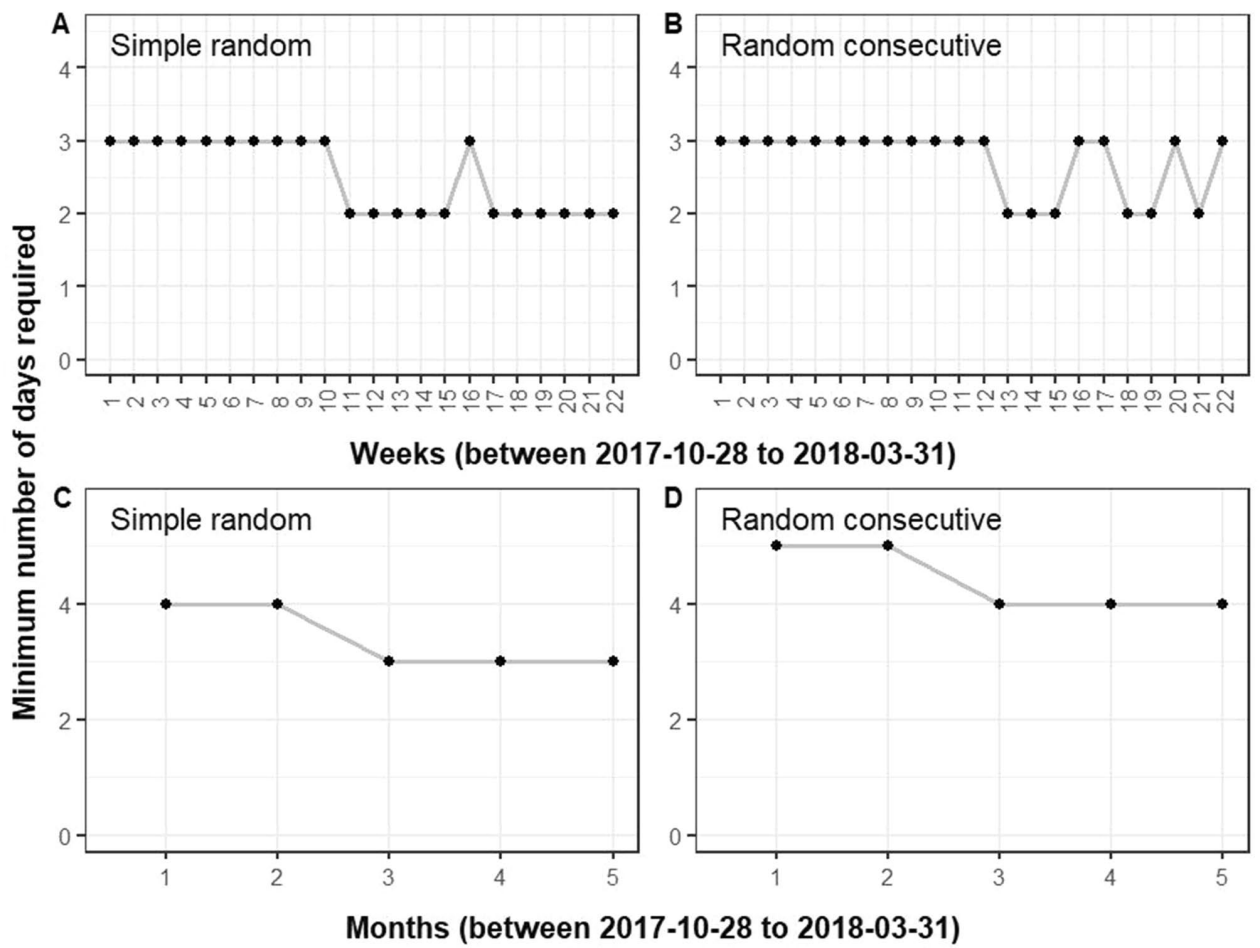
Minimum number of measurement days required to reliably estimate the mean daily step count for the 22 weekly time windows (N = 212,048) and five monthly time windows (N = 112,865) between 2017-10-28 and 2018-03-31. (**A**) simple random days in weekly time windows; (**B**) random consecutive days in weekly time windows; (**C**) simple random days in monthly time windows; (**D**) random consecutive days in monthly time windows. Figures were generated using R software version 3.6.1. (R Core Team (2020). R: A language and environment for statistical computing. R Foundation for Statistical Computing, Vienna, Austria. URL https://www.R-project.org/).

To achieve an ICC of 80% or above for reliable mean daily step count, the minimum number of measurement days is either 2 or 3 days for the 22 weekly time windows, regardless of the continuity of the days (Fig. 2). Table 2 shows that slightly fewer simple random days (mean 2.5, SD 0.5) are needed than random consecutive days (mean 2.7, SD 0.5). The difference was statistically significant (p-value = 0.025). When considering the minimum number of days, MAPE in daily step count was 14.5% and 14.6%, respectively, with and without restricting the continuity.

**Table 2:** Minimum number of days required to estimate weekly level of daily step count with ICC ≥ 80% and the corresponding MAPE (N = 212,048).

Characteristics	Simple random days		Random consecutive days	
	Minimum number of days	MAPE (%)	Minimum number of days	MAPE (%)
Total	2.5 (0.5)	14.6 (2.4)	2.7 (0.5)	14.5 (1.9)
**Nationality**	p-value = 0.157		p-value = 0.083	
Singaporean	2.5 (0.5)	14.3 (2.4)	2.7 (0.5)	14.1 (1.8)
Foreigner	2.6 (0.5)	14.9 (2.4)	2.9 (0.4)	14.6 (1.7)
**Gender**	p-value = 1.000		p-value = 0.046	
Female	2.5 (0.5)	14.9 (2.5)	2.7 (0.5)	14.9 (1.9)
Male	2.5 (0.5)	14.3 (2.3)	2.9 (0.4)	13.5 (1.5)
**Age (years)**	p-value < 0.001		p-value < 0.001	
17–39	3.0 (< 0.1)	13.7 (0.7)	3.0 (< 0.1)	14.7 (0.9)
40–59	2.5 (0.5)	14.2 (2.4)	2.6 (0.5)	14.4 (2.0)
60–79	2.0 (< 0.1)	13.7 (0.8)	2.1 (0.3)	13.9 (1.0)
80 +	2.0 (< 0.1)	12.4 (0.9)	2.0 (0.2)	12.7 (1.1)
**Body mass index (kg/m**^**2**^**)**	p-value = 0.008		p-value < 0.001	
< 18.5	2.3 (0.5)	15.9 (2.3)	2.6 (0.5)	15.5 (2.1)
18.5 to < 23	2.5 (0.5)	14.4 (2.2)	2.8 (0.4)	14.2 (1.7)
23 to < 27.5	2.5 (0.5)	14.4 (2.3)	2.7 (0.5)	14.2 (1.8)
≥ 27.5	2.6 (0.5)	14.6 (2.3)	3.0 (< 0.1)	13.7 (0.8)
**Participation of previous program season**	p-value = 0.317		p-value = 0.317	
No	2.5 (0.5)	15.0 (2.2)	2.7 (0.5)	14.8 (1.7)
Yes	2.5 (0.5)	14.1 (2.3)	2.8 (0.4)	13.9 (1.7)
**Step tracking device**	p-value < 0.001		p-value < 0.001	
HPB tracker 1	2.3 (0.5)	14.0 (1.8)	2.5 (0.5)	13.5 (1.7)
HPB tracker 2	2.0 (< 0.1)	13.1 (0.9)	2.0 (< 0.1)	13.7 (1.0)
HPB tracker 3	2.6 (0.5)	8.6 (2.2)	2.7 (0.5)	8.8 (2.2)
HPB tracker 4	2.6 (0.5)	10.4 (1.9)	2.8 (0.4)	10.3 (1.5)
iPhone	3.0 (0.2)	15.0 (1.1)	3.5 (0.5)	14.2 (1.9)
Samsung phone	3.0 (< 0.1)	14.7 (0.8)	3.5 (0.5)	14.0 (2.0)
Fitbit	3.0 (< 0.1)	11.3 (0.4)	3.0 (0.0)	12.1 (0.6)
Actxa	2.4 (0.5)	13.1 (2.0)	2.5 (0.5)	13.1 (2.2)

Data are in mean (SD) of results over 22 separate weeks. p-values were from Friedman's tests to compare minimum number of days required between participant groups (Supplementary Table S1).

*ICC* intraclass correlation coefficients, *MAPE* mean absolute percentage errors, *HPB* Health Promotion Board.

Minimum of 3 or 4 measurement days (mean 3.4, SD 0.5) are needed to reliably estimate the 5 monthly mean daily step count when using simple random days, while at least 4 or 5 days (mean 4.4, SD 0.5) are needed when using random consecutive days (Fig. 2, Table 3, Supplementary Figs. S1, S2). The difference was statistically significant (p-value = 0.025). On average, MAPE of mean daily step count is 13.4 (SD 1.5) % for simple random days and 12.9 (SD 1.1) % for random consecutive days, when using the minimum number of measurement days required.

**Table 3:** Minimum number of days required to estimate monthly level of daily step count with ICC ≥ 80% and the corresponding MAPE (N = 112,865).

Characteristics	Simple random days		Random consecutive days	
	Minimum number of days	MAPE (%)	Minimum number of days	MAPE (%)
Total	3.4 (0.5)	13.4 (1.5)	4.4 (0.5)	12.9 (1.1)
**Nationality**	p-value = 1.000		p-value = 1.000	
Singaporean	3.4 (0.5)	13.1 (1.5)	4.4 (0.5)	12.6 (1.0)
Foreigner	3.4 (0.5)	14.1 (1.7)	4.4 (0.5)	13.4 (1.3)
**Gender**	p-value = 1.000		p-value = 1.000	
Female	3.4 (0.5)	13.6 (1.6)	4.4 (0.5)	13.1 (1.1)
Male	3.4 (0.5)	13.1 (1.4)	4.4 (0.5)	12.5 (1.0)
**Age (years)**	p-value = 0.005		p-value = 0.005	
17–39	4.2 (0.4)	13.3 (0.8)	5.4 (0.5)	12.9 (1.0)
40–59	3.4 (0.5)	13.2 (1.5)	4.2 (0.8)	13.0 (1.9)
60–79	3.0 (< 0.1)	11.5 (0.7)	3.4 (0.5)	12.0 (1.4)
80+	2.4 (0.5)	11.8 (1.6)	2.8 (0.8)	11.9 (1.7)
**Body mass index (kg/m**^**2**^**)**	p-value = 0.392		p-value = 0.112	
< 18.5	3.2 (0.4)	14.1 (1.2)	4.0 (1.0)	14.0 (2.0)
18.5 to < 23	3.4 (0.5)	13.5 (1.5)	4.4 (0.5)	12.9 (1.2)
23 to < 27.5	3.4 (0.5)	13.2 (1.4)	4.4 (0.5)	12.7 (1.0)
≥ 27.5	3.4 (0.5)	13.6 (1.5)	4.4 (0.5)	13.2 (1.1)
**Participation of previous program season**	p-value = 1.000		p-value = 1.000	
No	3.4 (0.5)	13.7 (1.3)	4.4 (0.5)	13.1 (1.0)
Yes	3.4 (0.5)	13.3 (1.6)	4.4 (0.5)	12.7 (1.2)
**Step tracking device**	p-value < 0.001		p-value = 0.003	
HPB tracker 1	3.4 (0.5)	11.6 (1.1)	4.2 (0.8)	11.6 (1.3)
HPB tracker 2	2.6 (0.5)	11.8 (2.4)	2.8 (0.4)	12.3 (2.0)
HPB tracker 3	3.6 (0.5)	8.2 (1.7)	4.2 (0.8)	8.2 (1.8)
HPB tracker 4	3.4 (0.5)	9.7 (0.9)	4.2 (0.8)	9.7 (1.3)
iPhone	5.0 (0.7)	13.6 (1.1)	6.4 (1.1)	13.1 (1.0)
Samsung phone	4.6 (0.9)	13.7 (1.5)	6.0 (1.4)	13.1 (1.5)
Fitbit	4.4 (0.5)	10.6 (0.6)	5.4 (1.1)	10.4 (1.0)
Actxa	3.0 (0.7)	12.1 (1.7)	3.6 (0.5)	12.0 (1.4)

Data are in mean (SD) of results over five separate months. p-values were from Friedman's tests to compare minimum number of days required between participant groups (Supplementary Table S1).

*ICC* intraclass correlation coefficients, *MAPE* mean absolute percentage errors, *HPB* Health Promotion Board.

Consistent for both weekly and monthly time windows and both simple random days and random consecutive days, the minimum number of days required were larger in younger participants and those using smartphones or Fitbit to track step count (Tables 2, 3). The MAPE was larger among these participant groups even when more measurement days were used. In addition, for weekly time windows, being obese (body mass index ≥ 27.5 kg/m^2^) was associated with more minimum number of days for both simple random days and random consecutive days, while males require only more minimum random consecutive measurement days.

Sensitivity analysis shows that applying the same random days across participants does not affect the minimum number of days required (Table 4). Moreover, the minimum number of days required for weekly time windows do not change when the analysis included only the 112,865 participants who recoded daily step count for at least one complete month. Yet, fewer measurement days are needed among the 8185 participants who recorded daily step count on all the 155 days of the program intervention period.

**Table 4:** Minimum number of days required for weekly and monthly level of daily step count with ICC ≥ 80% and the corresponding MAPE, under different settings.

Settings	Simple random days		Random consecutive days	
	Minimum number of days	MAPE (%)	Minimum number of days	MAPE (%)
**Weekly time window**				
All participants, the same calendar days in all participants (N = 212,048)	2.5 (0.5)	14.3 (2.5)	2.6 (0.5)	14.8 (2.1)
Participants who recorded step count for at least one complete month (N = 112,865)	2.5 (0.5)	13.8 (2.4)	2.7 (0.5)	13.8 (2.0)
Participants who recorded step count for all 155 days (N = 8185)	2.0 (0.2)	13.7 (1.0)	2.1 (0.4)	14.0 (1.6)
Participants who recorded daily step count prior to the intervention (N = 44,469)	3.0 (0.0)	14.4 (0.6)	3.0 (0.0)	15.0 (0.5)
**Monthly time window**				
All participants, the same calendar days in all participants (N = 212,048)	3.4 (0.5)	13.5 (1.4)	4.2 (1.3)	13.2 (1.8)
Participants who recorded step count for at least one complete month (N = 112,865)	3.4 (0.5)	13.4 (1.5)	4.4 (0.5)	12.9 (1.1)
Participants who recorded step count for all 155 days (N = 8185)	3.0 (0.0)	13.0 (0.5)	3.4 (0.5)	13.4 (1.1)
Participants who recorded daily step count prior to the intervention (N = 16,460)	3.3 (0.6)	15.9 (1.2)	3.7 (0.6)	15.8 (0.5)

Data are in mean (SD) of results over 22 separate weeks or five separate months.

*ICC* intraclass correlation coefficients, *MAPE* mean absolute percentage errors.

## Discussion

Our study investigated the number of measurement days for reliable estimates of weekly and monthly mean daily step count levels, leveraging objectively measured daily step count from 212,048 adults spanning over 5 months using up to date trackers and smartphones. Overall, when there is no restriction regarding the continuity of the measurement days, 3 days weekly and 4 days monthly are sufficient for the corresponding time windows. For consecutive measurement days, a minimum of 3 and 5 days are needed to reflect weekly and monthly step count, respectively. More measurement days are required for reliable estimates in participants with younger ages and those using smartphone-based step count tracking (vs. wrist-worn trackers).

Together with the six studies identified by a systematic review of reviews in 2018, we found nine previous studies examining the day-to-day variability in stepping behaviour and the number of days needed to reliably estimate the daily step count level in adults^8–17^. Most studies have a time window up to 28 days, except for two small studies conducted over 365 days which found the minimal number of measurement days being 5–28 days for achieving ICC over 80%. A range of 2–4 days minimally were concluded by the four studies that investigated time windows of a week or shorter. The studies with 21- and 28-day time windows reported that a minimum of 5 and 7 days are adequate, respectively. The wide variability in findings of these limited number of studies may be due to the difference in measurement protocol, analytical approach, and study population. Small sample size and short measurement period in some studies may also contribute to the different findings, since limited statistical power leads to imprecise estimates. Some of the previous findings may also be biased because of violations of statistical assumptions such as the parallel tests conditions when using the Spearman–Brown prophecy formula. In comparison, our study analysed the variability of stepping behaviour in a large and diverse population independently on 22 weekly and 5 monthly time windows. Our findings are robust over time under each study setting. While consecutive measurements require an extra day for monthly time windows, overall, a minimum of 3 days for a week and 5 days for a month allow reliable estimation of mean daily step counts using both random and consecutive measurement days. The corresponding absolute measurement errors are consistently below 15%, although this is higher than sometimes recommended 10% cut-offs^16^.

Contrary to past studies which were all based on traditional hip-worn pedometers or research-grade accelerometers, our study appears to be the first investigating the day-to-day stepping variability using recent wrist-worn step trackers and smartphones with in-built accelerometers^9–17^. Enabled by the rapid technology advancement, wrist-worn fitness trackers and smartphone are becoming more and more widely adopted and integrated into everyday life. They have become common devices in step count based health practice and research^5–7^. Our study, therefore, addresses an important gap of measurement methodology in physical activity.

Our study found a significant difference in the required number of measurement days between participants using different step tracking devices. Compared with those using separate wrist-worn tracking devices, participants who used iPhones and Samsung phones directly to track step count consistently showed less stable day-to-day step count and required more measurement days for reliable estimates. The absolute errors in mean daily step count, assessed by MAPE, were also larger in smartphone users despite more measurement days used. Various factors could contribute to this observation, such as technical device characteristics, wearing location, and participant’s preference for tracking devices and physical activity behaviour. Dedicated investigations are warranted to elucidate the detailed relationships. Nevertheless, the significant difference found in our study indicates that future studies involving these devices would benefit from accounting for these differences.

Volume and intensity of physical activity are well known to differ by participant characteristics, such as age, gender, and weight status^27^. In comparison, less is known regarding the day-to-day variability, especially in daily step count^5,28^. Albeit scarce and heterogenous, past studies suggest that daily step count tends to be more stable among older adults^8–17^. Our findings agree with previous evidence. We found that older adults 60+ years require fewer measurement days, 2 days per week and four per month, to meet reliability criteria. Retiring from work might contribute to the lower day-to-day variability in stepping behaviour^5^. There was one previous study looking into gender difference. While the study of 81 elderly Japanese found that males need substantially more measurement days^9^, we only observed a very small gender difference for weekly time windows when requiring consecutive measurement days. Notably, for weekly time windows, participants’ body mass index was negatively associated with the minimum number of measurement days required.

Despite its strengths, our study also has several important limitations. First, our study subjects are participants of a population-wide physical activity program and are not representative of the entire adult population in Singapore. However, our large study population allows for sub-group analysis by several key participant characteristics. Secondly, our data were collected during an incentivised physical activity program, during which the variability of stepping behaviour may differ from typical free-living contexts. We repeated the analysis using participants’ daily step count prior to the program intervention period. The results of this sensitivity analysis demonstrated that the findings in this study are robust. Thirdly, the ICC values in this study are point estimates and the uncertainty has not been taken into account strictly. Due to the large data, it was too computationally intensive to repeat the random sampling and linear mixed effect modelling process for plenty times (e.g., 10,000 times) to obtain confidence intervals for the ICC values practically. However, the resulted minimum number of days from 5 repetitions of the process were the same, which indicated the robust results. Additionally, available data do not allow us to control the instrument variability within the same device model. The non-randomised study nature also limits the investigation of factors affecting the variation in day-to-day stepping behaviour.

In sum, we recommend at least three measurement days for a week and five measurement days for a month in adults to evaluate the mean daily step count level reliably using newly developed step tracking devices. While older adults age 60+ years may require only two measurement days weekly and four days monthly, we suggest 4 days weekly and 6 days monthly in individuals or studies tracking daily step via smartphones. Future studies on factors that influence the day-to-day variability of daily stepping measure, as well as their interaction effects, may foster the establishment of best practices in step count measurement and strengthen step count based health research.

## Supplementary Information

Supplementary Information.

## Data Availability

Data may be obtained from a third party (Health Promotion Board Singapore) and are not publicly available. The R-code could be obtained from the research team via the corresponding author.
