# Supplementary material for: Number of daily measurements needed to estimate habitual step count levels using wrist-worn trackers and smartphones in 212,048 adults
Source: Sci Rep. 2021 May 5;11:9633. doi: 10.1038/s41598-021-89141-3 (PMC8100112; doi:10.1038/s41598-021-89141-3)
Supplement: Supplementary file 1 — Supplementary Information. [file 41598_2021_89141_MOESM1_ESM.docx]

**Appendix**

This is appendix with supplementary materials for the manuscript entitled “Number of daily measurements needed to estimate habitual step count levels using wrist-worn trackers and smartphones in 212,048 adults”

**Content**

1. Figure S1: Intraclass correlation coefficients (ICC) for different number of measurement days for the 22 weekly time windows (N = 212,048) and five monthly time windows (N = 112,865) between 2017-10-28 and 2018-03-31.
2. Figure S2: Mean absolute percent error (MAPE) for different number of measurement days for the 22 weekly time windows (N = 212,048) and five monthly time windows (N = 112,865) between 2017-10-28 and 2018-03-31.
3. Table S1: Test statistics of Friedman's tests on group difference in minimum number of days required to estimate weekly and monthly level of daily step count.


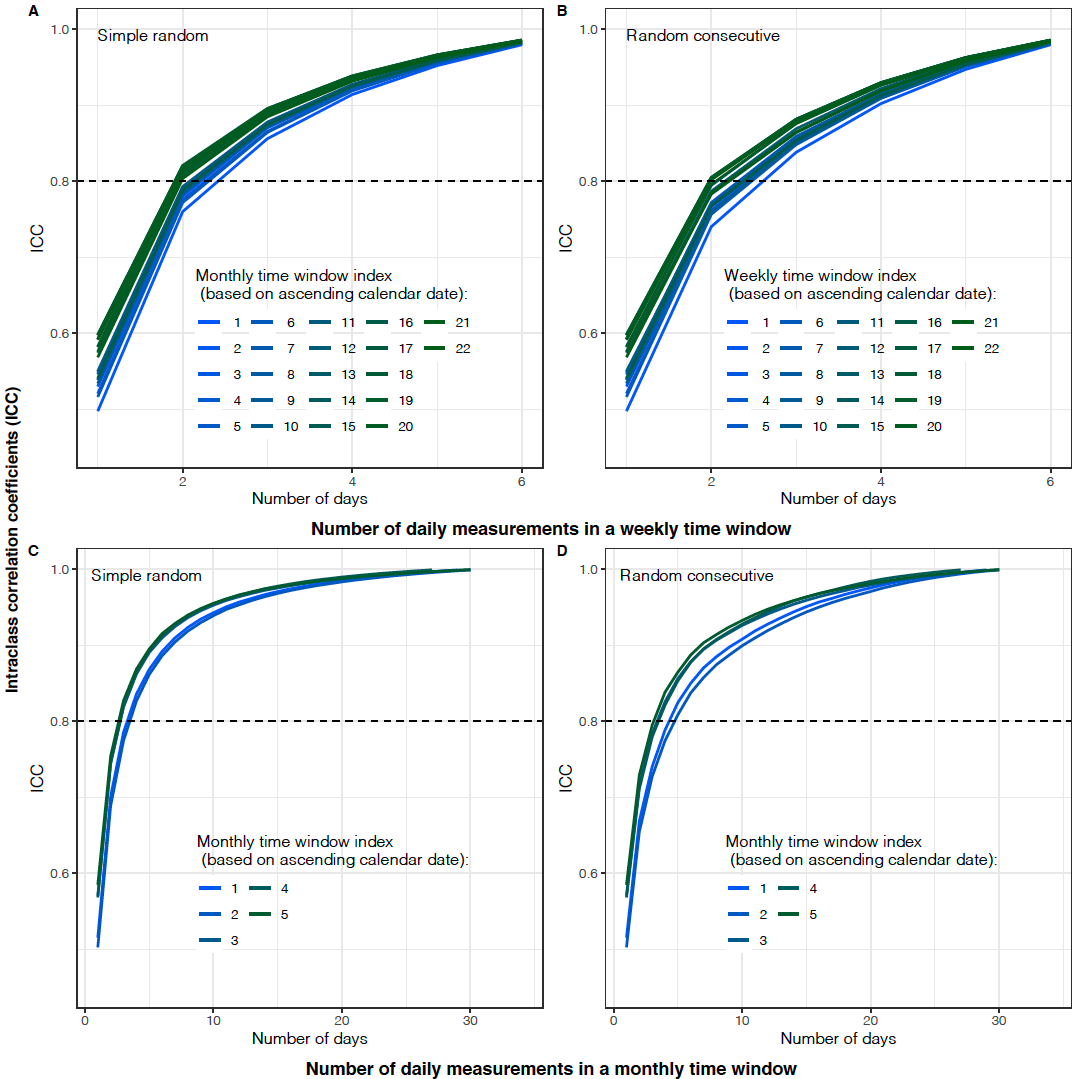


Figure S1: Intraclass correlation coefficients (ICC) for different number of measurement days for the 22 weekly time windows (N = 212,048) and five monthly time windows (N = 112,865) between 2017-10-28 and 2018-03-31. A: simple random days in weekly time windows; B: random consecutive days in weekly time windows; C: simple random days in monthly time windows; D: random consecutive days in monthly time windows. Figures were generated using R software version 3.6.1. (R Core Team (2020). R: A language and environment for statistical computing. R Foundation for Statistical Computing, Vienna, Austria. URL https://www.R-project.org/).


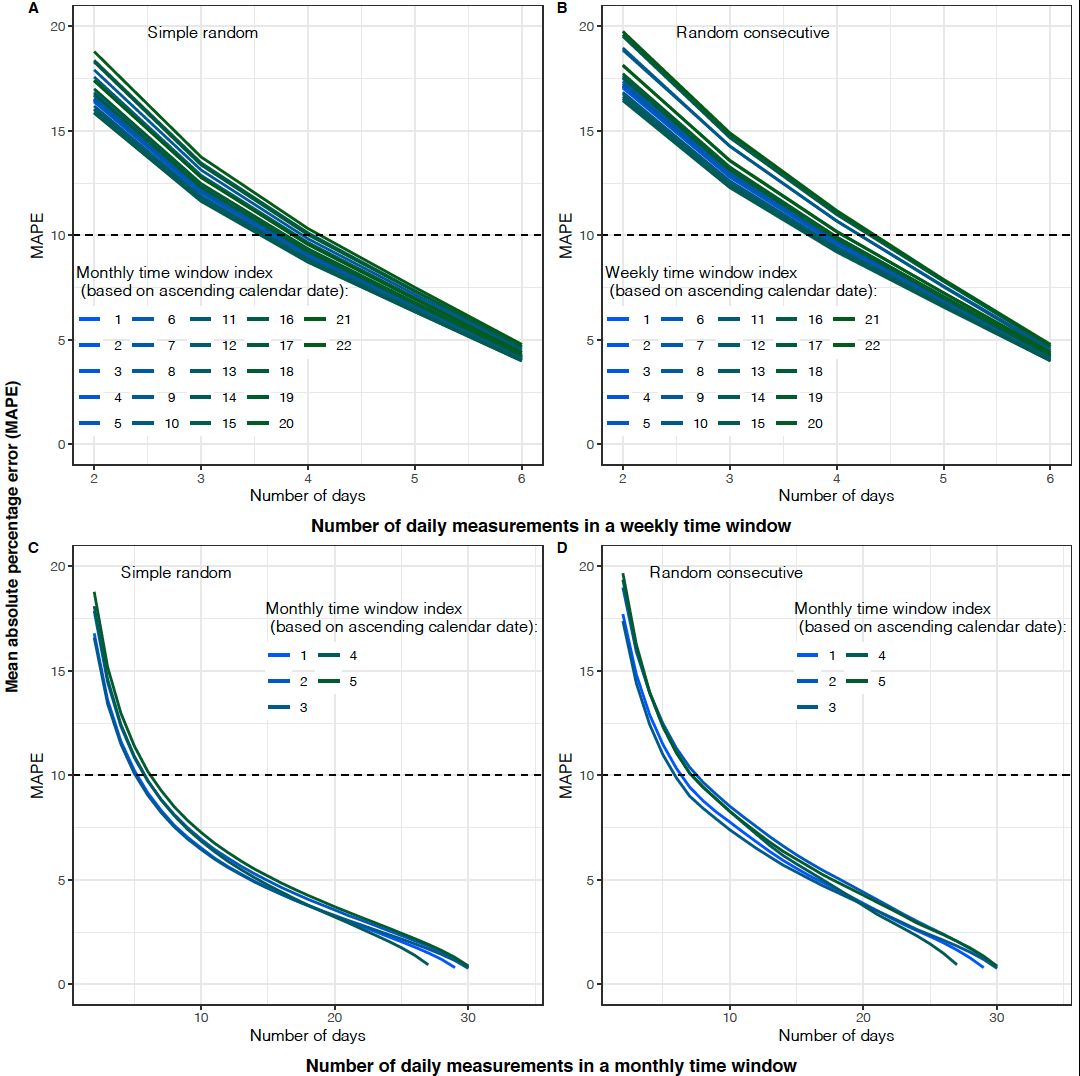


Figure S2: Mean absolute percent error (MAPE) for different number of measurement days for the 22 weekly time windows (N = 212,048) and five monthly time windows (N = 112,865) between 2017-10-28 and 2018-03-31. A: simple random days in weekly time windows; B: random consecutive days in weekly time windows; C: simple random days in monthly time windows; D: random consecutive days in monthly time windows. Figures were generated using R software version 3.6.1. (R Core Team (2020). R: A language and environment for statistical computing. R Foundation for Statistical Computing, Vienna, Austria. URL https://www.R-project.org/).

Table S1: Test statistics of Friedman's tests on group difference in minimum number of days required to estimate weekly and monthly level of daily step count.

| Time window type | Characteristics | Random days* | | | Random consecutive days* | | |
| --- | --- | --- | --- | --- | --- | --- | --- |
|  |  | Chi-Squared | Degree of freedom | p-value | Chi-Squared | Degree of freedom | p-value |
| Weekly | Nationality | 2.000 | 1 | 0.157 | 3.000 | 1 | 0.083 |
|  | Gender | -** | 1 | 1.000 | 4.000 | 1 | 0.046 |
|  | Age (year-old) | 51.857 | 3 | <0.001 | 48.760 | 3 | <0.001 |
|  | BMI (kg/m2) | 11.857 | 3 | 0.008 | 18.000 | 3 | <0.001 |
|  | Previous NSC participation status | 1.000 | 1 | 0.317 | 1.000 | 1 | 0.317 |
|  | Wearable | 97.480 | 7 | <0.001 | 110.103 | 7 | <0.001 |
| Monthly | Nationality | - | 1 | 1.000 | - | 1 | 1.000 |
|  | Gender | - | 1 | 1.000 | - | 1 | 1.000 |
|  | Age (year-old) | 12.857 | 3 | 0.005 | 12.938 | 3 | 0.005 |
|  | BMI (kg/m2) | 3.000 | 3 | 0.392 | 6.000 | 3 | 0.112 |
|  | Previous NSC participation status | - | 1 | 1.000 | - | 1 | 1.000 |
|  | Wearable | 31.921 | 7 | <0.001 | 32.413 | 7 | <0.001 |

*For both weekly and monthly time windows, the test statistics of Friedman's tests on difference in minimum number of days required between random days and random consecutive days are: Chi-Squared = 5; degree of freedom = 1; p-value = 0.0253.

**Chi-Squared values were unavailable and denoted as ‘-‘, when there was no group difference or the groups differed by a constant value consistently.
